# Assessing a novel way to measure step count while walking using a custom mobile phone application

**DOI:** 10.1371/journal.pone.0206828

**Published:** 2018-11-06

**Authors:** Christopher P. Hurt, Donald H. Lein, Christian R. Smith, Jeffrey R. Curtis, Andrew O. Westfall, Jonathan Cortis, Clayton Rice, James H. Willig

**Affiliations:** 1 Department of Physical Therapy, University of Alabama at Birmingham, Birmingham, Alabama, United States of America; 2 School of Medicine, University of Alabama at Birmingham, Birmingham, Alabama, United States of America; 3 Division of Clinical Immunology and Rheumatology, University of Alabama at Birmingham, Birmingham, Alabama, United States of America; 4 Department of Biostatistics, School of Public Health, University of Alabama at Birmingham, Birmingham, Alabama, United States of America; 5 CGI Group Inc, Montreal, Quebec, Canada; 6 Division of Infectious Disease, University of Alabama at Birmingham, Birmingham, Alabama, United States of America; Cardiff University, UNITED KINGDOM

## Abstract

**Introduction:**

Walking speed has been associated with many clinical outcomes (e.g., frailty, mortality, joint replacement need, etc.). Accurately measuring walking speed (stride length x step count/time) typically requires significant clinician/staff time or a gait lab with specialized equipment (i.e., electronic timers or motion capture). In the present study, our goal was to measure “step count” via smartphones through novel software and to compare with step tracking software that come standard with iOS and Android smartphones as a first step in walking speed measurement.

**Methods:**

A separate calibration and validation data collection was performed. Individuals in the calibration collection (n = 5) walked 20m at normal and slow speed (<1.0 m/s). Appropriate settings for the novel mobile application were chosen to measure step count. Individuals in the validation (n = 52) collection walked at 6m, 10m, and 20m at normal and slow walking speeds. We compared step difference (absolute difference) from observed step counts to native step tracking software and our novel software derived step counts. We used generalized estimated equation adjusted (participant level) negative binomial regression models of absolute step difference from observed step counts, to determine optimal settings (calibration) and subsequently to gauge performance of the shake algorithm settings and native step tracking software across different distances and speeds (validation).

**Results:**

For iOS/iPhone 6, when compared to observed step count, the shake service (software driven approach) significantly outperformed the embedded native step tracking software across all distances at slow speed, and for short distance (6m) at normal speed. On the Android phone, the shake service outperformed the native step tracking software at slow speed at 6 meters and 20 meters, while its performance eclipsed the native step tracking software only at 20 meters at normal speed.

**Discussion:**

Our software based approach outperformed native step tracking software across various speeds and distances and carries the advantage of having adjustable measurement parameters that can be further optimized for specific medical conditions. Such software applications will provide an effective way to capture standardized data across multiple commercial smartphone devices, facilitating the future capture of walking speed and other clinically important performance parameters that will influence clinical and home care in the era of value based care.

## Introduction

Walking speed is a reliable and valid measure, used as a prognostic and diagnostic indicator for heath and function. Walking speed is associated with mobility disability, mortality, and determining optimal rehabilitation care settings post hospital stay [1–3]. Some have even considered walking speed a sixth vital sign [4]. However, this measure is not widely used in typical clinical settings, and its capture is typically limited to research laboratories [5, 6]. Due to its powerful diagnostic and prognostic value and ease of capture, walking speed can be recorded by health professionals in clinical settings, or by nonprofessionals in home settings, facilitated with technology. This would provide important data to aid clinical decision making. In addition, such data recorded in a home environment and remotely monitored by a health professional could provide important, actionable insights into chronic disease management.

Biosensor-based health tracking devices are increasingly being used by the lay public and researchers to track health, mobility and fitness data [7–9]. While the ownership of such fitness trackers has grown in the US over the last decade, current and forecasted ownership of smart phones far outpaces increases in fitness tracker device uptake. Nearly two-thirds of Americans reported they owned smartphones in 2015 [10, 11] a number forecasted to continue to grow in the United States (US) in the near future [11, 12]. While fitness trackers provide several advantages, such as being lightweight and configurable to any body part, the fact that many people already own a smartphone makes it a reasonable platform to develop clinically relevant tests of walking.

Walking speed is the product of step length and step frequency (step count/time). Typically, mobile application and activity trackers record the number of “events” detected (i.e., steps) and display as step count. Accurately measuring step count is the first step to quantifying step frequency (i.e., step count/time). Step count, whether collected by a smartphone or fitness tracker, is reliable and valid when data are collected in a controlled environment on a treadmill and over a large number of steps [7, 13, 14]. However, smartphones and fitness tracker devices are much less accurate in over ground and free-living conditions [15]. When compared to observed step count, fitness trackers had an absolute mean step count differences from 0.3% to 9.6% when ambulating on a treadmill [13, 14]. Kooiman et al found that the criterion validity for monitoring step count in free-living condition with a commercially available smartphone application was 37.6% as measured by the mean absolute percentage error from the gold standard [14]. If mobile applications are going to be used to successfully measure rehabilitation outcomes overground, in clinical settings and homes, further refinement of the technology is required.

The time or distance walked may also affect the accuracy of counting steps with fitness trackers. Most normed rehabilitation measures only require 6 to 20 meters of space to measure walking speed [16, 17]. Typically, greater distances and walking times have been used in measuring the validity and reliability for fitness trackers. In fact, shorter walking distances/less steps has resulted in greater measuring error for these devices [13–15] that is also affected by walking speed [7]. Other studies support that pedometer applications for smartphones are much less accurate overground and when individuals walk slower than a comfortable pace [18]. Many smartphones come with native pedometer applications, however, similar to commercially available applications, the native pedometer application on iOS devices was much less accurate at slow walking speeds (<1.38m/s) and during overground conditions [19]. Greater error at shorter distances while walking overground and with slow walking speeds is problematic for the clinical utility of using smart phone applications to assess common clinical tests of walking speed. Our long-term goal is to provide clinical utility for tools that measure walking speed for clinicians at the point of care and to patients in their homes for longitudinal follow-up with minimal expense and greater accuracy over shorter ditances and slower speeds. Thus, rather than focusing on fitness tracker devices, we sought to test approaches to measure step count as a first step toward walking speed measurement via the more ubiquitous smartphones. In the present study, our purpose was to design and test a novel application to measure step count and to compare these results to those reported by health tracking applications that come standard with iOS (i.e., Apple Health Application) and Android (i.e., Google Fit) smartphones across different speeds and distances.

## Methods

### Setting

This study took place at the University of Alabama at Birmingham. The Institutional Review Board at the University of Alabama at Birmingham (UAB) approved the study protocol, and all participants provided written informed consent prior to study enrollment (UAB IRB protocol #X151202005).

### Overall study design

We developed a software-based method to count steps using the open source JavaScript plugin called shake.js available at GitHub (https://github.com/alexgibson/shake.js/). This plugin is designed to count events based on measured 3D accelerations of the phone. We tested this software based “shake service” approach versus native fitness tracking software embedded in sixth generation smartphones for iOS (iPhone 6, firmware 9.3.1) and Android (Nexus 6p, firmware 6.0.1). The shake service allows for adjustment of amplitude required to register as an event, or step in our case (i.e., sensitivity). We could also modify the refresh rate or refractory period, which was how often the software could count an event or step in our case. The study consisted of two phases. During the first study phase (Calibration), we calibrated the shake service for the mobile application, finding optimal settings to measure step counts at normal (> 1 meter/second) and slow (< 1 meter/second) walking speeds. The slow speed condition was added to assess the effect of walking speed on the calibration of the device. In the second phase of this study (Validation) we validated how well the shake service-based mobile application measured step count compared to observed step count and the native fitness tracking software found in the tested iOS and Android smartphones. We used the same two phones throughout the study.

### Smartphone calibration phase

First, we performed a calibration study to determine appropriate parameters for use with the shake service algorithm to most accurately detect steps at two ambulation speeds. Five individuals completed the phone calibration phase (25–54 years old, 1.67–1.87 m tall). We used individuals on the research team for the phone calibration phase as a convenience sample.

**Fig 1:**
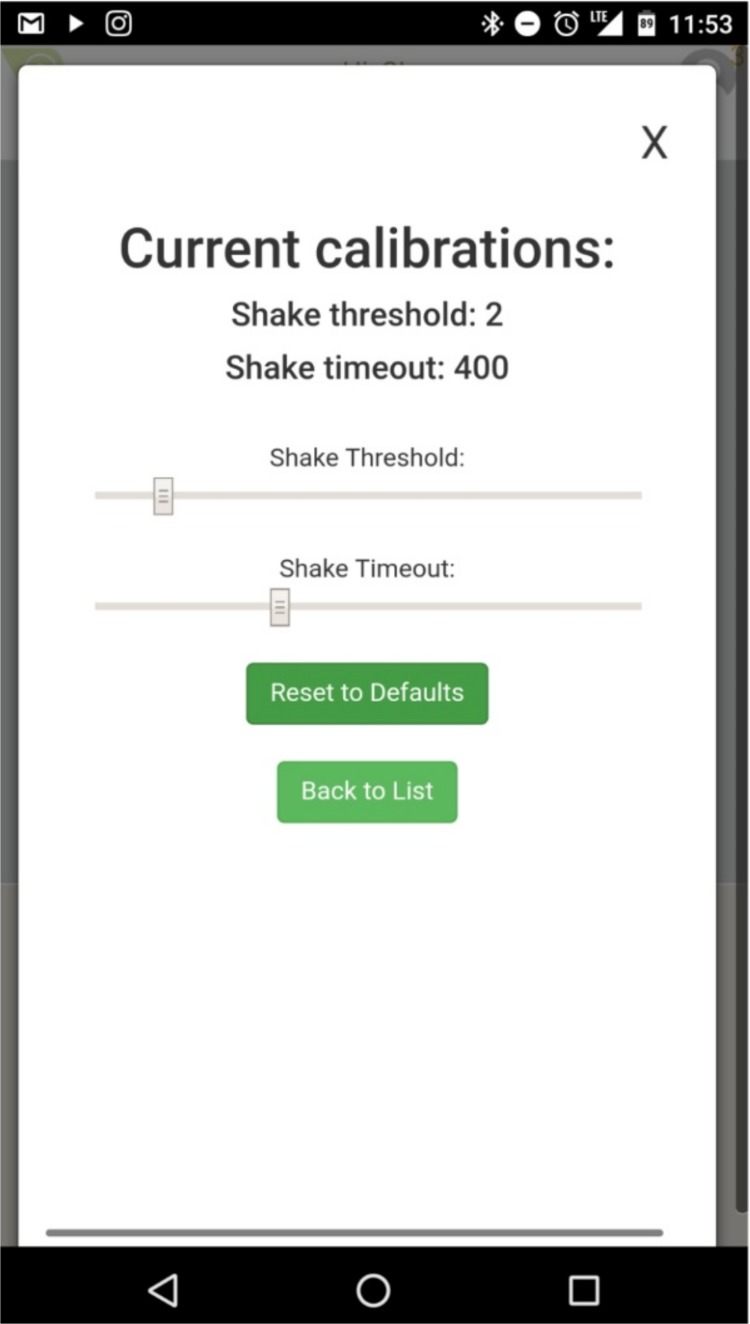
The interface for modifying shake service parameters is shown. The shake threshold relates to the amplitude required to register as an event and shake timeout which provided a window where a single event could be counted.

The shake service allows two parameters to be adjusted, sensitivity and refresh time. The shake algorithm has 19 different sensitivity settings and 19 different refresh times (Fig 1). During pilot testing, the investigators found that many of the combinations of sensitivity and refresh time were highly inaccurate. Further, we observed that different combinations of sensitivity and refresh time were required at different speeds. This makes sense biomechanically because of the positive linear relationship between walking speed and the vertical oscillations of the center of mass [20].

To limit the number of possible combinations, we first narrowed down the available options by observationally determining the nine best combinations of settings for each speed. The two speeds were labeled as normal (> 1 meter/sec) and slow (< 1 meter/second) pace. We tested the following combinations at a normal pace (sensitivity/refresh time): 1.5/400, 1.5/450, 1.5/500, 2.0/400, 2.0/500, 2.5/400, 2.5/450, and 2.5/500. Then, we tested the following combinations of sensitivity/refresh time at a slow pace: 1.0/400, 1.0/450, 1.0/500, 1.5/450, 1.5/500, 2.0/400, 2.0/450, 2.0/500.

To test each setting, we marked a 20-meter course with timing gates (Farmtek, Wylie, TX) at the start and end of the course. The timing gates were used to ensure that an individual was walking at a normal or slow speed. A trial was repeated if the person did not ambulate at the required speed.

Participants placed the iOS and Android smartphone in each of their front pant pockets. The participant activated the smartphone shake service step counter by tapping their smartphone screens prior to walking. Thus, the assessment was ‘active’ in that it was triggered explicitly by participants, rather than passively monitoring mobility in the background. Three researchers independently counted the participant’s steps. We defined a step from heel strike to contralateral heel strike while walking. We instructed participants to come to an immediate stop on the step where they crossed the 20m line to ensure that a full step was counted. The researchers reported the number of self-counted steps prior to looking at the step count on both phones. If there was a discrepancy in the investigators’ counts, a consensus was reached and recorded prior to revealing the application step counts. Each individual ambulated 3 times for each combination of sensitivity/refresh time settings listed above for both walking speeds. For quality control purposes, we recorded the first 5 participant attempts via digital camcorder and reviewed the video to confirm the observed step count matched the researcher’s reported count 100% at both normal and slow speed attempts.

### Statistical analysis calibration phase

To determine which combination of sensitivity and refresh time settings resulted in the least amount of deviation from the observed step counts, we fit negative binomial regression models modeling the count of the absolute difference between the count of observed steps and the shake algorithm reported steps. Separate models were fit for each speed (normal, slow). Each model included the nine fixed combinations of sensitivity and refresh time as the independent variable. As we had observations clustered among the 5 testers, we used generalized estimating equations (GEE) with an exchangeable correlation structure to account for the potential within observer correlation. Average absolute step differences and incidence rate ratios (IRR) are reported. All analyses were performed with SAS v. 9.4 (SAS, Cary, NC).

### Validation phase

We recruited 52 participants for the Validation Phase of the study (28.3 ± 9.9 years old, 28 males, 69.3 ± 4.4 inches). A cross-sectional study using a repeated measures design was used to determine concurrent validation of the phone’s step counters with the investigators observed step count. We set the shake algorithm calibration settings for the Validation Phase based on the review of the Calibration Phase results. Individuals were eligible to participate in this study if they reported no health problems that affected their ability to walk and were aged 18 years or older. We recruited participants from the greater UAB community. The methods used in this phase for collecting step count was the same as those used in the calibration phase of this study. However, individuals also walked down a 6 and 10-meter walkway flanked by gait timers in addition to ambulating 20 meters. Researchers commonly use distances of 6–20 m to assess walking speed [17, 21]. Prior to Validation testing, we confirmed all distances with a standard tape measure. Each participant ambulated each distance 3 times at both normal and slow gait speeds. Three trials were collected to assess repeatability (not assessed in the current analysis). The order of speed and distance was randomly selected to avoid order bias.

### Statistical analysis validation phase

Negative binomial regression models were fit modeling the count of the absolute difference between the observed steps and the shake algorithm reported steps. Separate models were fit for each phone (Android and iOS) at each speed (normal and slow) at each distance (6m, 10m, and 20m) for a total of 12 separate models with each model including the measurement tool (shake service versus the standard pedometer application) as the independent variable. As we had observations clustered among the 52 participants, we used generalized estimating equations (GEE) with an exchangeable correlation structure to account for the potential within participant correlation. All analyses were performed with SAS v. 9.4.

## Results

### Calibration phase

We recorded 270 observations for calibration of normal speed and 270 separate observations for the calibration of slow speed.

**Fig 2:**
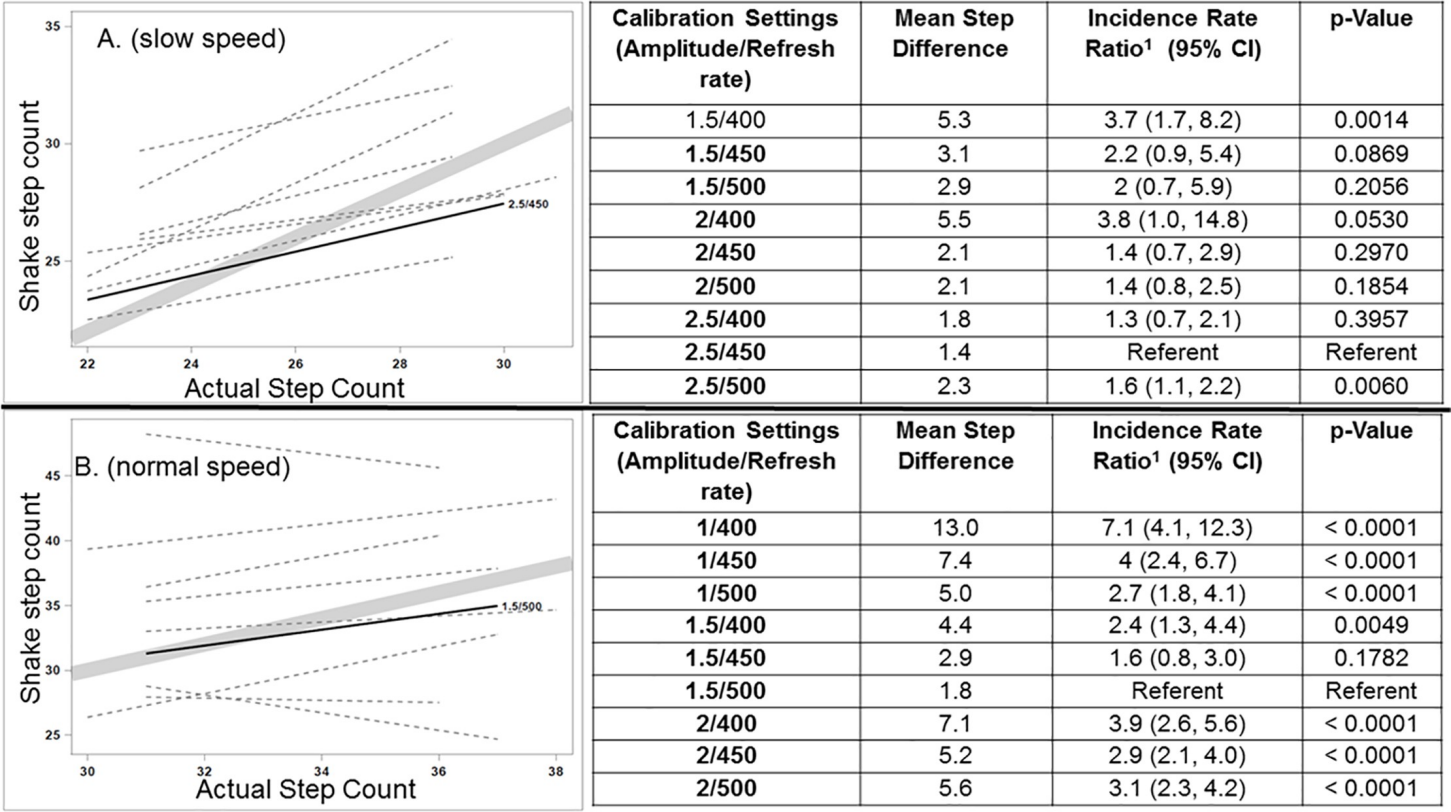
Calibration of Shake Algorithm across normal (2a) and slow (2b) speeds and models (name type) to determine the best fit to the observed step count line. For slow speed (2a.) 1.5/500 (sensitivity/refresh time) achieved the minimal step count difference from the observed step count (1.8 steps). For normal speed (2b) 2.5/450 (sensitivity/refresh time) achieved the minimal step count difference from the observed step count (1.4 steps).

For normal speed, the setting with the minimal step count difference from the observed step count was 2.5/450 (1.4 steps). In the negative binomial regression models, 2.5/450 was used as the referent and outperformed (p < 0.05) three settings (1.5/400, 2.0/400 and 2.5/500) and there was insufficient evidence to conclude any of the remaining settings were closer to the observed difference (no statistically significant difference observed, see Fig 2A).

For the 270 slow speed observations, the setting with the minimal step count difference from the observed step count was 1.5/500 (1.8 steps). In the negative binomial regression models, 1.5/500 outperformed (p < 0.05) all other settings, and there was insufficient evidence to conclude that the remaining setting (1.5/450) was closer to the observed difference (no statistically significant difference observed, see Fig 2B).

Thus, we calibrated the shake algorithm on both phones (iOS and Android) at 2.5/450 for normal speed and 1.5/500 for slow ambulation speed.

### Validation phase

Individuals walked at 0.81 ± 0.09 m/s for the slow walking condition and 1.38± 0.16 m/s for normal speed (p<0.001) creating two different speeds to test this application.

For iOS/iPhone 6, when compared to observed step count, the shake service (software driven approach) significantly outperformed the embedded native step tracking software across all distances at slow speed, and for short distance (6m) at normal speed (see Table 1).

**Table 1:** Step difference^1^ from observed step count for the iOS smartphone for different speeds (slow, fast) and distances (6m, 10m, 20m) and GEE adjusted negative binomial regression model of absolute step difference.

	Slow Speed^2^ (n-153)		P-value^3^	Normal Speed^4^ (n = 156)		P-value^3^
	Shake	Native Software		Shake	Native Software	
**6m**	2.16±1.97	8.15±4.48	**<0.001**	1.93±1.60	5.44±3.82	**<0.001**
**10m**	2.85±2.77	5.38±4.84	**<0.001**	2.48±2.19	2.94±3.37	0.188
**20m**	5.22±5.22	7.41±7.89	**0.029**	4.32±3.87	5.24±3.14	0.112

**1.** Absolute off, no directionality, step difference (either over or under) observed step count.

**2.** Slow speed is < 1 meter/second

**3.** P-values are from a GEE adjusted (participant level) negative binomial regression model of absolute step difference.

**4.** Normal speed is >1 meter/second

On the Android phone, the shake service outperformed the native step tracking software at slow speed at 6 meters and 20 meters, while its performance eclipsed the native step tracking software only at 20 meters at normal speed (see Table 2). Furthermore, a similar reported error rate was observed for 6 and 10 m distances across both phones.

**Table 2:** Step difference^1^ from observed step count for the Android smartphone for different speeds (slow, fast) and distances (6m, 10m, 20m) and GEE adjusted negative binomial regression model of absolute step difference.

	Slow Speed^2^ (n-153)		P-value^3^	Normal Speed^4^ (n = 156)		P-value^3^
	Shake	Native Software		Shake	Native Software	
**6m**	2.15±1.92	2.92±2.88	**0.006**	1.79±1.61	1.85±2.28	0.870
**10m**	2.52±2.50	2.89±3.49	0.374	2.03±2.28	1.47±1.60	0.066
**20m**	4.34±4.57	3.01±3.78	**0.033**	3.47±4.41	1.35±1.35	**<0.001**

**1.** Absolute off, no directionality, step difference (either over or under) observed step count.

**2.** Slow speed is < 1 meter/second

**3.** P-values are from a GEE adjusted (participant level) negative binomial regression model of absolute step difference.

**4.** Normal speed is >1 meter/second

## Discussion

Accurately measuring step count is a key first step towards building a mobilie application that can reliably measure walking speed (stride length x step count/time). In the present study, we compared step counts measured through a novel software driven approach (shake algorithm application) to native software on iOS and Android phones across different distances (6m, 10m, 20m) and speeds (< 1 m/s and > 1 m/s). Notably, previous research has validated step counts using long distances that are often not physically possible for individuals whose mobility is restricted by chronic medical conditions [7, 13–15]. In this study, we found that our software driven approach outperformed the native step tracking software found in Android and iOS phones across many walking speed and distance parameters. Software based approaches like our mobile application that allow for adjustment (e.g., amplitude and refresh rate, Fig 1) permit additional flexibility that can enable calibration for different clinical conditions to maximize accurate step count capture beyond what “one size fits all” step tracking software included in commercial smartphones and fitness devices offer. Software based approaches will aid dissemination and utilization as potential users will be able to diminish cost and employ already owned smartphone devices to capture walking speed, versus the purchase of yet another commercial device. Furthermore, our application encourages active assessment as opposed to many applications that operate in the background and continuously measure behavior that may (i.e., walking performance over ground) or may not (i.e., stair walking, walking through a crowded room) be the behavior of interest or may not be measured accurately, (i.e., if device is carried in a bag or purse at the time of the measurement).

While the shake algorithm outperformed the step tracking software in both phones for most experimental conditions, it did not do so across all our experimental conditions. We also note that there was error in step count associated with all distances between phones and within groups when compared to the reported observed step counts. This may be due to our selected calibration settings, as they were chosen based on a single distance and the limited number of individuals (n = 5) that we assessed. While these methodological decisions were made to guide data collection in this investigation, it is important to note that the shake algorithm can be further individualized to ensure accurate measures within a specific movement pattern or to optimize data capture for specific medical conditions. Settings could also be further adjusted by additional information (e.g. physical function as assessment by questionnaire from the NIH PROMIS system) [22], and may perform better when specifically calibrated for the Android vs iOS phones. We can envision a future where it is possible to target shake algorithm settings for specific conditions or patients so that mobile applications such as ours could be tailored to yield even better results. This is a significant advantage of our novel software based approach versus commercial fitness tracker devices where settings may be more difficult to adjust and where hardware diverges amongst devices [13–15]. Due to being adaptable and adjustable, we believe the shake algorithm to provide a promising/sound direction to pursue further research and a great foundational step towards measuring walking speed in clinical settings and patient’s homes.

In terms of speed, for both phones the shake algorithm was more accurate than the native software when the participants walked less than 1 m/s (meter per second) for all observations except measurements by the Android phone at 20m. Previous investigators found that slower speeds increased step count error [7, 13]. Older adults and individuals with acute and chronic conditions tend to ambulate at a slower pace [3, 23–25]. The shake application performed well at 6-meters in both phones regardless of walking speed. This is advantageous for measurement in private residences where long straight distances may be scarce as many homes do not have more than 6 to 10 meters to ambulate without turning. This represents another reason our software driven approach may outperform native software found in current phones to measure step counts to subsequently derive walking speeds for future patient monitoring in home settings. Such monitoring seems poised to gain importance in the coming years as the medical field continues to stress the importance of home management of chronic illness to prevent hospitalization, a foundational pillar of population health and value based care initiatives [26].

While fitness trackers can include enhanced monitoring sensors for a variety of measures, the added cost and complexity of these devices may represent barriers to their widespread adoption and use. In contrast, the ubiquity of smartphones is growing [10, 12] at a greater pace than that of fitness trackers, making solutions that piggyback on this existing technology more attractive for dissemination to the general population. We posit, that mobile applications therefore may be the better technology to acheive remote monitoring of patients by healthcare workers due to its generalizability and published reports that almost half of health tracker owners quit wearing them within the first six months [27].

Our study has limitations. First, we tested this application on only two phones. Different generations and models of smart phones utilized different hardware, that may affect their measurement error. However, one important feature is that we can customize our software to optimize parameters to each participant regardless of phone hardware. Second, we elected to select one sensitivity/refresh time for each ambulation speed for both smartphones. There exists the possibility that we could have achieved even better results had we individualized settings to a specific phone. With this investigation we chose to demonstrate that one software solution could be used across devices with different operating systems (iOS, Android). However, we recognize that device specific settings or even condition or patient specific settings could be possible in the future, potentially further enhancing the performance of the mobile app. Third this study was performed in a healthy adult sample and the results that we saw with this population may not generalize to other populations. Our intent is to replicate this study and recalibrate our assessment parameters as needed in older patients with chronic illnesses. Fortunately, our approach would allow for customization across different medical conditions in the future. Finally, we need to test this algorithm for even longer distances to determine if it could be used for other functional measures such as the 6-minute walk test. Previous researchers stated that mobile applications tend to have less step count error the longer the distance walked, an effect that may very well further enhance the accuracy of our approach in future testing across longer distances [14, 15].

### Conclusions

The shake service based mobile application (software based approach) provides an effective way to standardize data capture across multiple available commercial smartphones as well as provides the option to customize settings for different medical conditions in the future. We will continue to explore this approach and build towards accurately measuring walking speed on commercial smartphones to facilitate access to this important clinical parameter to aid clinicians at the point of care. This technology could further assist patients as they monitor their progress in their homes during longitudinal follow-up in the age of population health and value based care.
